# Amitriptyline inhibits bronchoconstriction and directly promotes dilatation of the airways

**DOI:** 10.1186/s12931-023-02580-6

**Published:** 2023-10-31

**Authors:** Paulina Hempel, Virag Klein, Anna Michely, Svenja Böll, Annette D. Rieg, Jan Spillner, Till Braunschweig, Saskia von Stillfried, Norbert Wagner, Christian Martin, Klaus Tenbrock, Eva Verjans

**Affiliations:** 1 Department of Pediatrics, Medical Faculty, RWTH Aachen, University Hospital Aachen, Pauwelsstraße 30, 52074 Aachen, Germany; 2 Institute of Pharmacology and Toxicology, Medical Faculty, RWTH Aachen, University Hospital Aachen, Aachen, Germany; 3 Department of Anaesthesiology, Medical Faculty, RWTH Aachen, University Hospital Aachen, Aachen, Germany; 4 Department of Thoracic and Cardiovascular Surgery, Medical Faculty, RWTH Aachen, University Hospital Aachen, Aachen, Germany; 5 Institute of Pathology, Medical Faculty, RWTH Aachen, University Hospital Aachen, Aachen, Germany

**Keywords:** Amitriptyline, Bronchoconstriction, Bronchodilation, Inhibition, Muscarine receptor, PCLS, EFS, IPL, flexiVent

## Abstract

**Introduction:**

The standard therapy for bronchial asthma consists of combinations of acute (short-acting ß_2_-sympathomimetics) and, depending on the severity of disease, additional long-term treatment (including inhaled glucocorticoids, long-acting ß_2_-sympathomimetics, anticholinergics, anti-IL-4R antibodies). The antidepressant amitriptyline has been identified as a relevant down-regulator of immunological T_H_2-phenotype in asthma, acting—at least partially—through inhibition of acid sphingomyelinase (ASM), an enzyme involved in sphingolipid metabolism. Here, we investigated the non-immunological role of amitriptyline on acute bronchoconstriction, a main feature of airway hyperresponsiveness in asthmatic disease.

**Methods:**

After stimulation of precision cut lung slices (PCLS) from mice (wildtype and ASM-*knockout*), rats, guinea pigs and human lungs with mediators of bronchoconstriction (endogenous and exogenous acetylcholine, methacholine, serotonin, endothelin, histamine, thromboxane-receptor agonist U46619 and leukotriene LTD4, airway area was monitored in the absence of or with rising concentrations of amitriptyline. Airway dilatation was also investigated in rat PCLS by prior contraction induced by methacholine. As bronchodilators for maximal relaxation, we used IBMX (PDE inhibitor) and salbutamol (ß_2_-adrenergic agonist) and compared these effects with the impact of amitriptyline treatment. Isolated perfused lungs (IPL) of wildtype mice were treated with amitriptyline, administered via the vascular system (perfusate) or intratracheally as an inhalation. To this end, amitriptyline was nebulized via pariboy in-vivo and mice were ventilated with the flexiVent setup immediately after inhalation of amitriptyline with monitoring of lung function.

**Results:**

Our results show amitriptyline to be a potential inhibitor of bronchoconstriction, induced by exogenous or endogenous (EFS) acetylcholine, serotonin and histamine, in PCLS from various species. The effects of endothelin, thromboxane and leukotrienes could not be blocked. In acute bronchoconstriction, amitriptyline seems to act ASM-independent, because ASM-deficiency (Smdp1^−/−^) did not change the effect of acetylcholine on airway contraction. Systemic as well as inhaled amitriptyline ameliorated the resistance of IPL after acetylcholine provocation. With the flexiVent setup, we demonstrated that the acetylcholine-induced rise in central and tissue resistance was much more marked in untreated animals than in amitriptyline-treated ones. Additionally, we provide clear evidence that amitriptyline dilatates pre-contracted airways as effectively as a combination of typical bronchodilators such as IBMX and salbutamol.

**Conclusion:**

Amitriptyline is a drug of high potential, which inhibits acute bronchoconstriction and induces bronchodilatation in pre-contracted airways. It could be one of the first therapeutic agents in asthmatic disease to have powerful effects on the T_H_2-allergic phenotype and on acute airway hyperresponsiveness with bronchoconstriction, especially when inhaled.

**Supplementary Information:**

The online version contains supplementary material available at 10.1186/s12931-023-02580-6.

## Introduction

Bronchial asthma is a chronic inflammatory airway disease with increasing prevalence worldwide (up to 300 million people), in which first symptoms often arise in childhood [1, 2]. The high prevalence and chronic nature of asthmatic disease as well as its treatment over the long term present a serious economic burden to our society [3]. Current therapy is based on combinations of acute (short-acting ß_2_-adrenegic agonists) and, depending on severity of disease, additional long-term treatment (including inhaled glucocorticoids, long-acting ß_2_-sympathomimetics, anticholinergics, anti-IL-4R antibodies) [4]. New substances combining an amelioration of bronchoconstriction with inhibition of the allergic T_H_2 response are now needed.

In preliminary studies, we demonstrated that inhaled amitriptyline, an inhibitor of acid sphingomyelinase (ASM) and known as a tricyclic antidepressant, has immunomodulatory effects, such as a lowering of the production of typical T_H_2-cytokines in T lymphocytes of asthmatic mice (unpublished data). ASM-*knockout* as well as ASM-inhibition via amitriptyline resulted in an alleviated T_H_2-phenotype [5]. In a model of ovalbumin-induced asthma, Böll et al*.* demonstrated reduced bronchial hyperresponsiveness, lower numbers of eosinophils and typical T_H_2-lymphocytes in BAL fluid and lung tissue and lower levels of IL-4 and IL-5. Additionally, systemic administration of amitriptyline has been shown to lead to clinical improvement in asthmatics, but only in a very small cohort with 8 and 12 patients [6]. Studying the literature of subsequent years, amitriptyline appears to have been forgotten as an anti-asthmatic medication since new antidepressants with fewer side effects have become available [7]. Nevertheless, amitriptyline as an ASM-inhibitor did provide a beneficial inhalation therapy in patients with cystic fibrosis, with minimal adverse systemic effects [8]. Amitriptyline treatment in the clinical trials of Riethmueller et al*.* resulted in FEV1 improvement of CF lungs of up to 17%, which is in the same range as modern CFTR-modulator therapies [9, 10]**.**

Dyspnea caused by bronchoconstriction is perhaps the most frequent symptom of asthmatics after exposure to an asthmatic trigger. It is a tightening of airway smooth muscles surrounding the bronchi and bronchioles with consequent wheezing and shortness of breath [11]. Activation of airway smooth muscle cells by bronchoconstrictors results in a rapid rise in [Ca^2+^]_i_ through the release of calcium from intracellular stores [12]. The role of G-proteins, protein kinases and Ca^2+^, and molecular interaction within contracting filaments of muscle are well defined, but the exact mechanisms by which a wide range of stimuli initiate these events are not fully understood [11]. This airway narrowing is treated by short- and long-acting *β*_2_-agonists that operate through activation of the *β*_2_-adrenergic receptor. Antagonists of muscarinic acetylcholine receptor M3 are used to reduce bronchoconstriction by blocking the action of acetylcholine-induced pathways. In anti-asthmatic therapy, leukotriene antagonists that block the signaling of cysteinyl leukotriene receptor 1 are used as an add-on therapy to reduce ongoing bronchoconstriction and inflammation induced by cysteinyl leukotrienes [3].

Studies from the 1980s and earlier, used radioligand-binding assays to demonstrate a non-selective binding of amitriptyline to muscarine receptors of the brain [13, 14]. Additionally, amitriptyline seems to bind to serotonin receptors, but the effect of this binding is unknown [15]. Studies on the effect of amitriptyline on bronchoconstriction in the lung were no more than rudimentary. Dose-dependent amitriptyline treatment led to either bronchoconstriction (high concentration of 10 mM) or bronchodilatation (middle concentration of 50 µM) in single individual studies [16, 17].

In our study, we aimed to investigate whether amitriptyline influences acute bronchoconstriction directly and non-immunologically. Prior experiments had led to the hypothesis that this antidepressant is relevant as a pharmacological inhibitor of airway contraction, one of the main features of bronchial asthma. To address this question, we treated PCLS of wildtype C57/Bl6, ASM-*knockout* animals and fitting littermates with various mediators of bronchoconstriction, e.g., exogenous and endogenous acetylcholine, serotonin, histamine, thromboxane-receptor antagonist U46619 and leukotriene LTD4 in the presence or absence of amitriptyline and then calculated changes in airway area. We next focused on the effect of inhaled amitriptyline on lung function parameters in the model of isolated perfused lung (IPL) and mouse ventilation with the flexiVent setup. Thereafter, we investigated the anti-obstructive potential of amitriptyline on bronchodilatation following acute bronchoconstriction, since this situation resembles the typical exacerbation of acute bronchial asthma.

In summary, this study should help to evaluate the potential role of amitriptyline in the treatment of bronchial asthma based on a combination of its well-defined immunological und non-immunological effects.

## Material and methods

### Animals and human lung biopsies

Experiments were performed on 7–12 week-old C57Bl/6 wildtype (Charles river) and ASM-*knockout* (Smpd1^−/−^) mice and wildtype (Smpd1^+/+^) littermates (described previously [18]), 6–10 week-old rats (Wistar, Charles River), and 6–10 week-old guinea pigs (Dunkin Hartley, Charles River). The study was approved by the regional governmental authorities and animal procedures were performed according to the German animal protection law (AZ 81-02.04.2018.A095, A4-30332A4).

Human PCLS were prepared from patients undergoing lobectomy due to cancer. After pathological inspection, cancer free tissue was taken from the periphery of the lung. None of the patients showed any signs of pulmonary hypertension or asthmatic inflammation (echocardiographic or histological evaluation). The study was approved by the local ethics committee (EK 61/09) of the Medical Faculty Aachen, RWTH Aachen. All patients gave written informed consent.

### LDH cytotoxicity test

LDH activity in PCLS was determined in culture supernatants using the Cytotoxicity Detection Kit (LDH) obtained from Roche following manufacturer’s instructions. Amitriptyline with a concentration of 100 µM and triton-X 100 were used as positive controls.

### Precision cut lung slices

Precision cut lung slices (PCLS) were prepared as described previously [19, 20]. Briefly, intraperitoneal anesthesia in mice, guinea pigs and rats was performed with pentobarbital (Narcoren, Garbsen, Germany) and deepness of anesthesia was verified by missing reflexes and cardiac arrest. Thereafter, the trachea of rats and guinea pigs was cannulated and the abdomen and diaphragm were opened up. Human lungs were filled via a main or lobar bronchus. The lungs were filled with 1.5% low-melting point agarose (rats, human) or 8% gelatine (mice, guinea pigs) and cooled on ice. Tissue cores were prepared and cut into 250–300 µm thick slices with a Krumdieck tissue slicer (Alabama, Munford, AL, USA). PCLS were incubated at 37 °C and several medium changes were performed in order to wash out the agarose. Only slices free of agarose or gelatine, with beating cilia, intact smooth muscle layer and in a relaxed state were used for the subsequent experiments 24–48 h hours after preparation.

### Concentration response curves in PCLS

To show the effect of amitriptyline on bronchoconstriction, PCLS of rat, mouse, guinea pig and human lungs were preincubated for 30 min with 0 µM, 0.1 µM, 1 µM and 5 µM amitriptyline in a suspension with P/S-Medium (total 1000 µl). The PCLS were then treated with the following mediators of airway constriction to calculate concentration response curves:

**Table:** 

Mediator	Concentrations
Acetylcholine	10^–9^–10^–2^ M
Serotonin	10^–9^–10^–1^ M
Endothelin	10^–9^–10^–5^ M
U46619	10^–11^–10^–4^ M
Histamine	10^–9^–1 M
LTD4	10^–12^–10^–5^ M

During the experiment, the intraluminal area of the airways was monitored with a digital video camera (Leica Viscam 1280, Leica DFC 280 and USB cameras, Supervision). For measurement of bronchoconstriction, PCLS were placed into a culture dish and were fixed by a platinum wire with threads (teflon ring for EFS) to the bottom of the dish to avoid movement during measurement. Images were recorded every 5 s for a time period of 5 min until the next concentration was added. The images were analyzed with Optimas 6.5 (Media Cybernetics, Bothell, WA) and ImageJ software. The airway area before addition of the lowest concentration of the agonist was defined as 100%. Bronchoconstriction was expressed as a percentage of the initial airway area (IAA). GraphPad Prism 9 software (GraphPad Software, San Diego, CA, USA) was used for fitting sigmoidal concentration response curves.

### Induction of dilatation and recontraction in PCLS

For dilatation experiments, PCLS were stimulated with methacholine 10^−6^ M (longer lasting contraction than acetylcholine). Contraction was monitored for 20 min and all slices that had contracted to an area between 30 and 50% of the initial airway area were used for further investigations. A combination of IBMX (1 mM, dissolved in DMSO) and salbutamol (1 mM, dissolved in ethanol (EtOH)) was used as positive control for maximal dilatation. Amitriptyline 1 µM and 5 µM were tested, alone and in combination with IBMX/salbutamol. Saline, EtOH and DMSO were used as negative controls. Slices were incubated for a further 50 min and images were recorded every 10 s. For observation under USB microscopes (supervision) and constant temperature of the medium, PCLS were again fixed to the bottom of cell culture plates using a platinum wire. Images were analyzed taking the initial airway area as 100%.

After airway dilatation, we re-induced airway contraction with rising concentrations of methacholine (10^–5^–10^−3^ µM). The culture medium was not changed for these experiments and thus contained the same substances as before.

### Electric field stimulation in PCLS

EFS were performed as described before for rats [21]. Briefly, a PCLS was transferred to a cavity of a standard 12-well plate, placed between two platinum electrodes, weighted down by a teflon ring and 1 ml incubation medium was added. In preliminary experiments the following settings were identified as useful: train rhythm (TR) = 60 s, train width (TW) = 2.5 s, frequency (*F*) = 50 Hz, pulse duration (*B*) = 1 ms, current (*A*) = 200 mA [22]. The electric field was delivered by a Hugo Sachs Electronics Stimulator II (March-Hugstetten, Germany). Airways were monitored by videomicroscopy and airway area used once again for quantification. Each PCLS was exposed to four stimulation trains. The acetylcholine esterase inhibitor neostigmine (10 μM) was added 10 min before the start of the first train. Responses were again monitored at a frame rate of 0.2 s^−1^ for 5 min. Amitriptyline was added at 0.1 µM (2nd cycle), 1 µM (3rd cycle) and 5 µM (4th cycle).

### Isolated perfused lung

The mouse lungs were prepared and perfused essentially as described recently [23]. After preparation, the lungs were perfused for 30 min without any treatment in order to obtain a baseline. As perfusion medium, we used a gelafundin supplemented medium. 1 µM amitriptyline was added at t = 30 min (reflecting systemic administration).

In further experiments, 67 µg amitriptyline in 50 µl volume were administered intratracheally via a microsprayer (PennCentury, USA) at t = 30 min as inhaled treatment without systemic application. The lungs were ventilated by pressure of 8 cmH_2_O, a PEEP of 3 cm H2O with 90 breaths min^−1^ and a tidal volume of about 200 μl. Every 5 min a hyperinflation (− 20 cm H_2_O) was performed. All data were transmitted to a computer and analyzed by the Pulmodyn software (Hugo Sachs Elektronik, March Hugstetten, Germany). Concentration response curves were produced for the response to inhaled acetylcholine (10^–9^–10^−4^ M) and resistance parameters were measured. Concentrations of inhaled and systemic amitriptyline were calculated as close to equivalent in our experiments based on results of unpublished preliminary studies.

### Flexivent ventilation

All mice were exposed to nebulized amitriptyline (3.3 mg/ml) or saline 0.9% for 10 min in a nebulization chamber with a connected pariboy (nebulization with red insert, size of nebulization droplets: 2.8 µm) shortly before start of ventilation. After nebulization, mice were tracheotomized with a 20G cannula and connected to the ventilator. All mice were initially anaesthetized with pentobarbital sodium (70 mg/kg) and fentanyl (0.1 mg/kg). Anaesthesia was maintained with pentobarbital sodium (20 mg/kg) alone after 30 min. Lidocain (1%, 50 µl) was used as local anesthetic. All mice were mechanically ventilated with a tidal volume (Vt) of 10 ml/kg and a positive end-expiratory pressure (PEEP) of 2 cmH_2_O using the flexiVent (SCIREQ, Canada) ventilation setup. Body temperature was rectally controlled and adjusted to between 36.5 and 37.5 °C over the whole ventilation period. Continuous data recording of heart rate and ECG was performed to monitor cardiovascular function.

Dynamic lung mechanics were measured by applying a sinusoidal standardized breath and analyzed with forced oscillation technique. We used a 1.2 s, 2.5 Hz single-frequency forced oscillation manoeuvre (SnapShot perturbation) and a 3 s, broadband low frequency forced oscillation manoeuvre with 13 mutually prime frequencies of between 1 and 20.5 Hz (Quick Prime perturbation). Total lung resistance (Rrs) was calculated by the flexiVent software (flexiWare 7.0.1, SCIREQ, Canada) by fitting measured SnapShot values to the linear single compartment model using multiple linear regressions. Respiratory system input impedance was calculated from the QuickPrime data and tissue resistance (tissue damping, G) was assessed by iteratively fitting the constant-phase model to input impedance.

Following basal ventilation of 10–15 min until a stable baseline was reached, airway hyperresponsiveness was provoked with nebulized acetylcholine (Ach, 0–56.2 mg/ml, 10 µl) via an aeroneb nebulizer in the inspiratory line. For each concentration, lung function was measured 12 times (SnapShot and QuickPrime) over a period of 3 min. Every 5 min, short volume-controlled recruitment maneuvers (deep inspirations of over 3 s) were used to avoid atelectasis. After provocation of bronchial hyperresponsiveness, mice were sacrificed by exsanguination via the carotid artery.

### Statistical analysis

Quantitative results are expressed as means ± standard error of the mean (SEM). Statistical analyses of dose response curves obtained in PCLS experiments were performed by Schild regression analysis (GraphPad Prism 5.0). Statistics of lung function parameters generated in IPL and flexiVent experiments, electric field stimulation in PCLS and LDH measurements were carried out using the general linear mixed model analysis (PROC GLIMMIX, SAS9.4, SAS Institute Inc., Cary, United States), assuming a normal distribution except for LDH values, which have a log-normal distribution. Homoscedasticity was tested using the covtest statement. In case of heteroscedasticity of data, degrees of freedom were adjusted using the Kenward-Roger approximation. Multiple comparisons were corrected using the Shaffer-simulated (SIM) stepdown procedure. For dilation experiments different time points or treatments were analyzed by one-way ANOVA, with Tukey’s correction for multi comparison. A p-value of < 0.05 was considered statistically significant (*p < 0.05, **p < 0.01, ***p < 0.001).

## Results

**Fig. 1:**
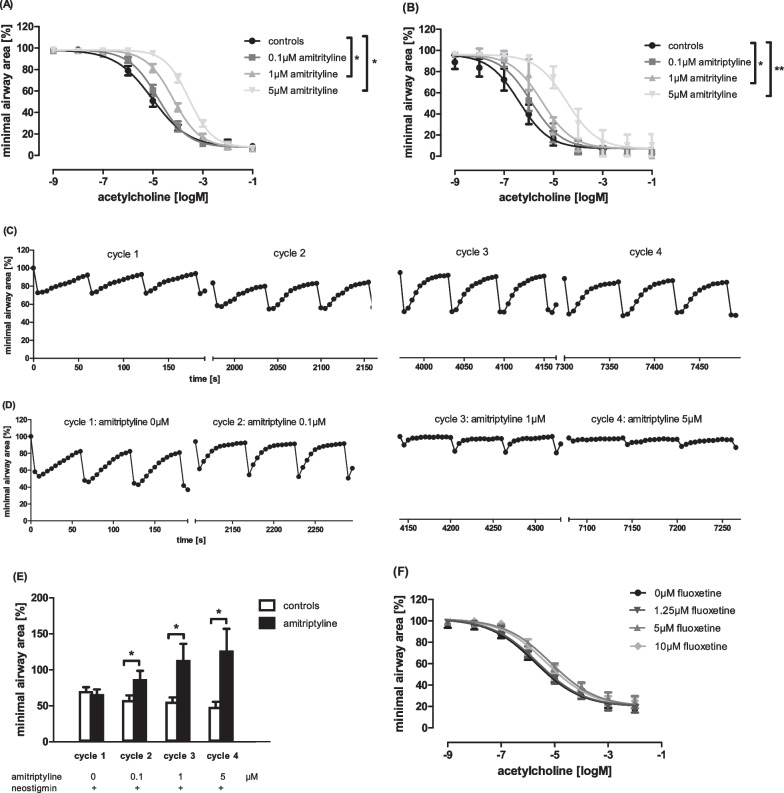
Amitriptyline inhibits acetylcholine-induced airway constriction. **A** Concentration–response curves of acetylcholine-stimulated PCLS of rats and **B** mice pre-treated with different concentrations of amitriptyline (0–5 µM). **C** Exemplary cycles of electric field stimulation (EFS) in rat PCLS with saline and **D** amitriptyline-treatment. Neostigmin was used as stabilisator of bronchoconstriction. **E** Minimal airway area in EFS experiments with amitriptyline. **F** Acetylcholine-induced airway constriction in mouse PCLS pre-treated with fluoxetine. **A**, **B** All groups n = 6. **E**, **F** Controls n = 4, all other groups n = 6. **A**, **B**, **E**, **F** All graphs represent means ± SEM; *p < 0.05, **p < 0.01

In previous studies, we demonstrated that ASM-*knockout* and ASM-blockade via amitriptyline inhibit the immunological T_H_2-phenotype in acute bronchial asthma (Böll et al. [5]). Our focus here was on the non-immunological, direct effect of amitriptyline on acute bronchoconstriction. First, PCLS of rats and mice were pre-incubated with amitriptyline in rising concentrations (0–5 µM) and airway constriction was induced by acetylcholine, which acts via the muscarinic acetylcholine receptors and is one of the main bronchoconstrictors in acute exacerbation of bronchial asthma. Controls without amitriptyline showed the typical dose-dependent reduction of airway area in PCLS of rats and mice, with a characteristic sigmoid-shaped curve (Fig. 1A, B). Pre-incubation with amitriptyline significantly attenuated acetylcholine induced bronchoconstriction in lungs of both species (Fig. 1A, B). This effect was dose-dependent and even more pronounced with amitriptyline 1 µM and 5 µM.

Thus, we were able to demonstrate the ameliorative effect of amitriptyline on bronchoconstriction induced with exogenously added acetylcholine. To verify that amitriptyline also decreases airway constriction caused by endogenous acetylcholine released through cholinergic nerves by means of electric field stimulation (EFS) in rat PCLS, the EFS-triggered contractions were increased through use of the acetylcholine esterase inhibitor neostigmine (10 μM). Without amitriptyline treatment, electric stimuli resulted in a more marked reduction of airway area than with amitriptyline pre-incubation in rising concentrations (Fig. 1C, D). Amitriptyline 5 µM almost completely inhibited airway contraction caused by stimulation of cholinergic synapses.

Several clinical studies and case reports document the potential toxicity of amitriptyline in different organs (e.g. [24–26]) but in those studies and cases, the lung was not the main organ of interest. Nonetheless, lactate dehydrogenase (LDH) toxicity tests in PCLS 1 h and 24 h after amitriptyline treatment showed no relevant changes in LDH levels (Additional file 1: Fig. S1A, B), indicating that there were no short-term toxic effects on lung tissue.

Amitriptyline, as well as fluoxetine, belong to a group of compounds known as functional inhibitors of the acid sphingomyelinase (FIASMA), which influence the ASM/ceramide system. In previous studies, the ceramide content of bronchial epithelial cells of mice with cystic fibrosis became normalized after inhalation with amitriptyline or fluoxetine [8]. Thus, we also wanted to investigate the role of fluoxetine, a SSRI and FIASMA, on direct bronchoconstriction. Interestingly, this antidepressant caused no significant inhibition of acetylcholine-induced airway contraction in the mouse PCLS (Fig. 1E). Therefore, we hypothesized that amitriptyline perhaps could work ASM-independent.

**Fig. 2:**
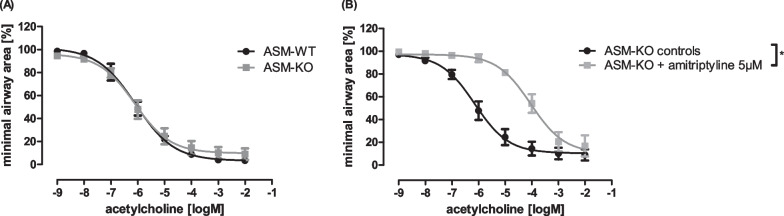
The effect of amitriptyline on bronchoconstriction is ASM-independent. **A** Concentration–response curves of PCLS of *ASM-knockout* and wildtype littermates stimulated with acetylcholine. **B** Concentration response curves of PCLS of ASM-*knockout* mice pre-treated with amitriptyline (5 µM). **A**, **B** n = 4 in all groups. Graphs represent mean ± SEM; *p < 0.05

As described previously, amitriptyline has immunomodulatory effects in bronchial asthma, stemming from an inhibition of the ASM [5]. In contrast, ASM-*knockout* did not ameliorate acute bronchoconstriction (Fig. 2A). Acetylcholine-induced airway contraction was the same in ASM-*knockout* and wildtype animals. Therefore, a complete inhibition of ASM did not influence airway hyperresponsiveness. To establish whether amitriptyline diminishes contraction of airways via the inhibition of ASM, we treated wildtype and ASM-*knockout* animals with acetylcholine in rising concentrations. If amitriptyline acts in an ASM-dependent manner, we would expect no effect of amitriptyline treatment in ASM-deficient mice. However, when we pre-incubated PCLS from ASM-*knockout* animals with 5 µM amitriptyline, we were able to demonstrate the potential anti-obstructive effect of amitriptyline (Fig. 2B). Amitriptyline does therefore appear to have a non-immunological, ASM-independent mechanism of action in the lung.

**Fig. 3:**
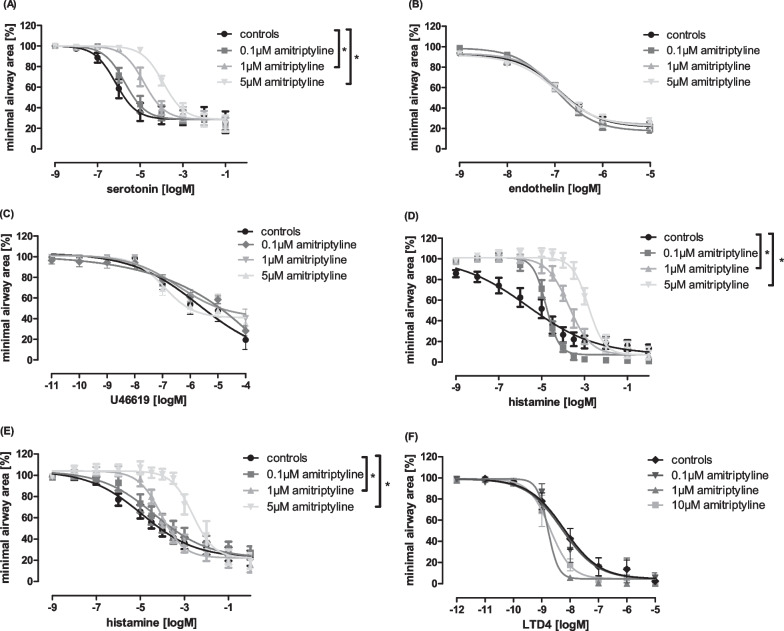
Amitriptyline clearly reduces the effect of different mediators of bronchoconstriction in rat, guinea pig and human PCLS. **A** Concentration–response curves of rat PCLS stimulated with serotonin, **B** endothelin and **C** U46619. **D** Concentration–response curves of PCLS of guinea-pigs and **E** humans stimulated with histamine. **F** Concentration–response curves of guinea-pig PCLS stimulated with LTD4. **A**–**F** Pre-incubation with amitriptyline in rising concentrations. Rats: n = 6 in all groups, guinea pigs: n = 7 in all groups, humans: controls and Ami 1 µM: n = 6; Ami 0.1 and 5 µM: n = 9. All graphs represent means ± SEM; *p < 0.05

Next, we investigated whether amitriptyline could also diminish the potential of further mediators of airway constriction. While incubation of rat PCLS with amitriptyline strongly decreased serotonin-induced airway contraction, we could not find any effect on bronchoconstriction mediated by endothelin or thromboxane A2 mimetic U46619 (Fig. 3A–C). It is known from the literature that histamine and leukotrienes are the main mediators of airway contraction in humans and guinea pigs, but in rats these mediators have no relevant effect [27]. We therefore induced bronchoconstriction in amitriptyline pre-treated PCLS of human lungs and of guinea pigs and demonstrated strong and significant inhibition of contraction compared to controls (Fig. 3D, E). Bronchoconstriction induced by leukotriene LTD4 in guinea pigs could not be inhibited by amitriptyline treatment (Fig. 3F). All experiments showed a stronger effect of amitriptyline at 5 µM compared to lower concentrations.

In summary, amitriptyline clearly inhibits ASM-independent muscarinic, serotonergic and histaminergic acute bronchoconstriction in the lung.

**Fig. 4:**
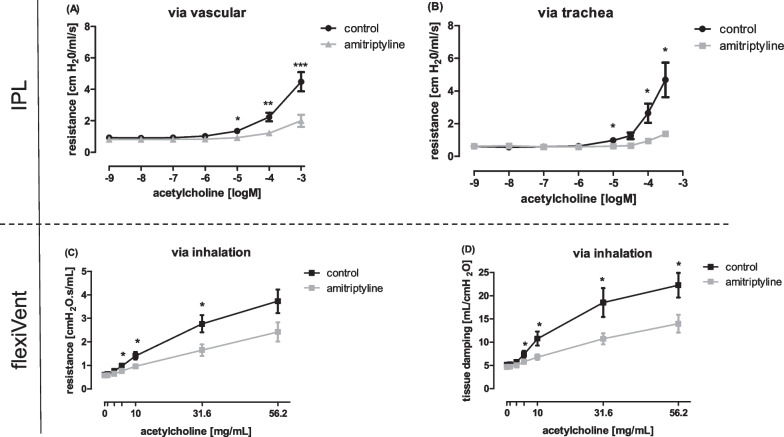
Amitriptyline strongly improves lung function in obstructive lung disease. **A** Resistance in mouse IPL after systemic or **B** intratracheal treatment with amitriptyline (1 µM Ami in perfusat medium and 67 µg in 50 µl intratracheally). **C** Total resistance and **D** tissue resistance in wildtype mice after pre-inhalation with amitriptyline and following ventilation with the flexiVent setup. **A**, **C**, **D** n = 6 in both groups.** B** n = 4 in both groups. All graphs represent means ± SEM; *p < 0.05, ***p < 0.001

To verify the relevance of therapeutic amitriptyline in lung disease, we next focused on the role of systemic and inhaled amitriptyline in mouse ventilation models. First, isolated perfused lungs of mice (IPL) were ventilated with a tidal volume of about 200 μl. The lungs were perfused via a cannulation in the pulmonary artery with 1 µM amitriptyline in normal perfusate. This setup reflects systemic administration of amitriptyline (Fig. 4A). Acute bronchoconstriction was induced with acetylcholine in rising concentrations. Control mice showed a typical increase in airway resistance, whereas that of amitriptyline-treated animals rose less dramatic. On intratracheal administration of amitriptyline with a microsprayer, reflecting inhalation therapy, the difference in airway resistance was stronger, reaching a mean of 4.68 cm H_2_O/ml/s in controls compared to 1.37 cm H_2_O/ml/s in amitriptyline-treated animals (Fig. 4B).

For our in-vivo model, all mice were exposed to nebulized amitriptyline or saline 0.9% for 10 min in a chamber with connected pariboy shortly before start of ventilation. After pre-treatment, mice were anaesthetized and ventilated with a tidal volume (Vt) of 10 ml/kg and a positive end-expiratory pressure (PEEP) of 2cmH_2_O using the flexiVent ventilation setup. Bronchial hyperresponsiveness was again tested with acetylcholine in rising concentrations administered via an aeroneb nebulizer in the inspiratory line. Amitriptyline-treated animals displayed significantly better lung function parameters compared to controls (Abb. 4C-D). Total Resistance (R), mainly determined by the state of the central airways, and tissue resistance (tissue damping) were clearly better in animals pre-treated with amitriptyline than in controls. Hence, airway hyperresponsiveness was significantly attenuated through a single inhalation of amitriptyline, which was conducted shortly before start of ventilation.

In summary, we were able to show, both in-vitro and in-vivo, that amitriptyline has an anti-obstructive effect in acute bronchoconstriction. This effect extends to the main pathways of airway contraction subject to muscarinic, serotonergic and histaminergic stimulation. Both, systemic administration and inhalation of amitriptyline clearly inhibited airway hyperresponsiveness.

**Fig. 5:**
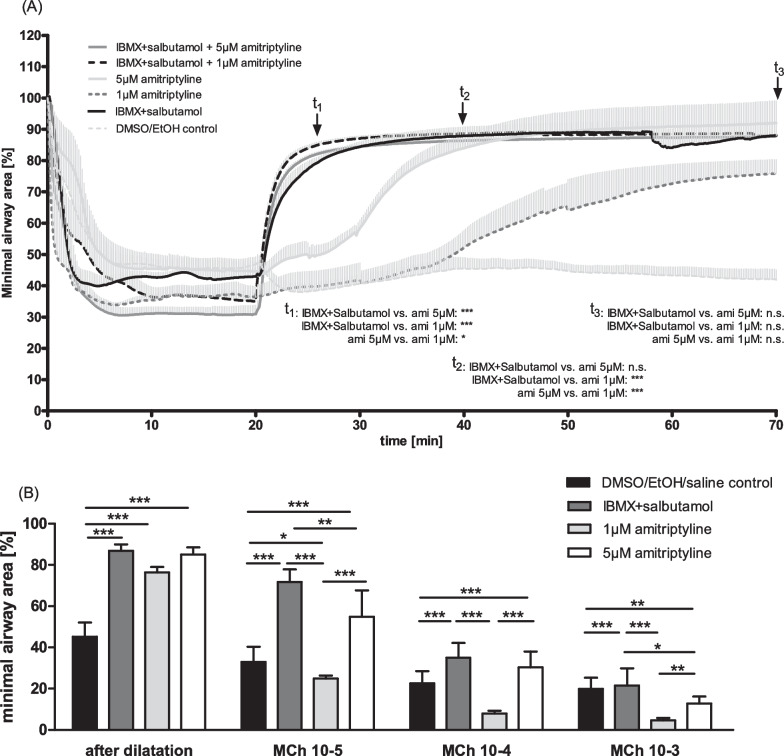
Amitriptyline causes bronchodilatation in obstructive lung disease. **A** Pre-contraction of rat PCLS with methacholine, following dilatation with IBMX/salbutamol, amitriptyline and combination therapy. Minimal airway area. n = 6 in all groups. **B** Re-contraction of dilated PCLS with rising concentrations of methacholine. Airway area after dilatation and following stimulations with methacholine. n = 4 in all groups. All graphs represent means ± SEM; *p < 0.05, **p < 0.01, ***p < 0.001

We next investigated the effect of amitriptyline on the dilatation of pre-contracted airways. This situation better reflects the typical clinical scenario with an acute obstruction of the airways, where drugs are needed to reopen the bronchi and bronchioles, in particular. For these experiments, we induced long-lasting airway contraction in PCLS with methacholine (10^−6^ M). All slices showing an airway area of 30–50% of the initial value were stimulated with the mediators IBMX/salbutamol, amitriptyline 1 µM and 5 µM (Fig. 5A). As controls, contracted PCLS were treated with saline, DMSO or ethanol, which produced exactly the same results. The combination of IBMX and salbutamol was used as a potent mix of mediators for maximal dilatation. In controls with saline/DMSO/ethanol no relevant change in airway area was observed, while IBMX/salbutamol induced rapid dilatation to approximately 90% of the initial airway area. Treatment with amitriptyline 5 µM also had a strong anti-obstructive effect, which occurred later than that of IBMX/salbutamol. Low 1 µM concentrations of amitriptyline still led to a dilatation, up to 75% of initial airway area, but the slope of this curve was not as steep as that seen with amitriptyline 5 µM or the rapid increase after treatment with IBMX/salbutamol (see different significances for amitriptyline 5 and 1 µM compared to IBMX/salbutamol with and without amitriptyline at t_1_ = 25 min, t_2_ = 40 min und t_3_ = 70 min). Combining IBMX/salbutamol with amitriptyline 1 µM or 5 µM did not improve dilatation of the airways any further (Additional file 1: Fig. S2).

When we recontracted these slices—after bronchodilation—with rising concentrations of methacholine, the combination of IBMX/salbutamol and amitriptyline 5 µM was most effective at inhibiting airway constriction, followed by IBMX/salbutamol + amitriptyline 1 µM (Fig. 5B, Supplemental Additional file 1: Fig. S2A). Amitriptyline 5 µM alone and IBMX/salbutamol could prevent airway contraction really effectively, but even low-dose (1 µM) amitriptyline alone had a relevant and significant effect. Thus, amitriptyline clearly induces dilatation following acute bronchoconstriction.

## Discussion

The evidence presented here shows amitriptyline to be a potential inhibitor of bronchoconstriction and a promising agent for bronchodilatation of pre-contracted airways. Amitriptyline clearly decreases the degree of airway contraction induced by exogenous or endogenous (EFS) acetylcholine, serotonin and histamine in PCLS from different species. However, the effects of thromboxane and leukotriene could not be blocked by this well-known antidepressant. We clearly demonstrated that amitriptyline acts ASM-independent in acute bronchoconstriction. Using the model of isolated perfused lungs (IPL), we were able to show that both systemic and inhaled amitriptyline improve ventilation after acetylcholine provocation of airway constriction. In the flexiVent setup, we ventilated mice that had been treated with inhaled amitriptyline directly prior to ventilation and demonstrated that acetylcholine-induced bronchial hyperresponsiveness was much stronger in untreated animals than in treated ones. Additionally, we obtained clear evidence that amitriptyline not only inhibits bronchoconstriction but also dilatates pre-contracted airways, in some cases more effectively than typical bronchodilators such as IBMX as PDE inhibitor and salbutamol as ß_2_-agonists. In summary, we have demonstrated in tissue from a number of species including human lungs that both systemically administered and inhaled amitriptyline present a sound basis for potential new strategies in airway diseases involving bronchoconstriction. Furthermore, the drug could be applied as prophylactic, long-term or acute-onset medication.

Amitriptyline was the second tricyclic antidepressant to appear on the market for a major depressive disorder in 1961 [28]. In the brain, it inhibits serotonin and norepinephrine reuptake and exerts its effect at cerebral histaminergic and muscarinic receptors or on downstream pathways [29, 30]. Later on, amitriptyline therapy was used for a range of disorders such as chronic pain, sleep disorders or anxiety. In the 1960s to 1980s, several studies demonstrated the anti-histaminergic, anti-muscarine and anti-serotonin activity of this medication (e.g. [31, 32]), mainly in the brain. Radioligand binding analysis showed non-selective binding of amitriptyline to the muscarinic acetylcholine receptor in the brain and in other organs [30, 33], but the M3 receptor, which is mainly expressed in the lung, was not investigated in detail (e.g. [34]). As anti-muscarinic medication, systemically administered amitriptyline led to typical anticholinergic side effects, such as cconstipation, dry mouth, dry eyes, urinary retention and so on (e.g. [35]). These effects paved the way for new, more selective and highly compatible antidepressants, like SSRIs and SNRIs.

Only very few studies described the effect of amitriptyline on bronchoconstriction or airway inflammation. Matsunaga et al*.* showed that smooth muscle reactivity in isolated tracheal rings from rats was attenuated by amitriptyline incubation [36]. However, the concentrations of amitriptyline used by these authors in their study (> 1 µM) were in the higher µM range and partially toxic. The authors concluded that amitriptyline plays a role in the inhibition of phosphatidylinositol, the Ca^2+^-calmodulin-myosin light chain pathway and possibly also in the Rho-kinase pathway. In summary, it was difficult to identify a single target directly influenced by amitriptyline binding that caused various downstream changes. In contrast to our data, Dahlin et al*.* published experiments with isolated perfused lungs, which were exposed to 100 µM amitriptyline via the pulmonary circulation [17]. These amitriptyline-perfused lungs showed higher airway resistance through bronchoconstriction and additional constriction of related vessels mediated by higher concentrations of endothelin-1. In line with these data, Svens et al*.* demonstrated that endothelin as well as platelet activating factor and protein kinase activation are important mediators of the amitriptyline-induced impairment of lung function in isolated perfused lungs [37]. This was the first study to reveal possible dose-dependent effects of amitriptyline with bronchoconstrictive effects at doses > 50 µM. We used much lower amitriptyline concentrations of 0.1 to 5 µM. The drug was evaluated as non-toxic in toxicity tests and we were able to demonstrate a clear inhibition of bronchoconstriction. The toxic effects of high-dose amitriptyline are, however, relevant because these effects lead to cell stress and cell death. In our experiments on mice, inhalations with amitriptyline produced serum levels much below the toxic range and approximately 30–50% of normal serum levels after systemic intake of low-dose amitriptyline in humans (unpublished data). Interestingly, we demonstrated an inhibitory effect of amitriptyline on bronchoconstriction induced by acetylcholine/methacholine, serotonin or histamine, but no effect on the thromboxane U46619- or leukotriene LTD4-induced decrease in airway area. The structures of all corresponding receptors are very different, so that it is hard to imagine amitriptyline inhibiting their action through direct binding in each case. From further studies it is well known that amitriptyline can bind to muscarinic acetylcholine and histamine receptors, but the resulting effects are not investigated in detail yet [38]. Further studies should therefore focus on the precise effect of amitriptyline within the signalling pathway of relevant receptors. Once the mechanism of amitriptyline’s positive effect has been clarified, potential targets for treatment of bronchial asthma can be investigated and developed.

Since amitriptyline is one of the drugs with the longest history of clinical use, it could potentially become a cheap and easily accessible medication for patients suffering from diseases with acute bronchoconstriction, mainly bronchial asthma. A clinical trial with patients with cystic fibrosis demonstrated a beneficial effect of amitriptyline on FEV1 increase (up to 17%) with only mild and mostly transient side effects [10]. Inhalation of amitriptyline in a mouse model was without systemic effects and resulted in reduced ceramide concentrations in the lung [8]. Up to now, this drug has never been used for inhalation in clinical trials.

Amitriptyline is known to be a functional inhibitor of the acid sphingomyelinase. ASM is involved in the regulation of the typical T_H_2-phenotype in allergic asthma [5, 39]. In one of these studies, we demonstrated that ASM-*knockout* results in a reduced asthmatic reaction of ovalbumin-sensitized animals through a lowering of the numbers of eosinophils, T_H_2-lymphocytes and typical T_H_2-cytokines. Hence, the ASM clearly influences the immunological phenotype in asthmatic airway disease. In the present study, PCLS from ASM-*knockout* and wildtype mice showed comparable degrees of airway contraction after stimulation with acetylcholine. Nevertheless, pre-treatment with amitriptyline induced a clear inhibition of airway contraction, also in ASM-*knockout* animals. Fluoxetine, as additional well-known ASM-inhibitor, did not inhibit acute airway contraction. Thus, in acute obstruction, the effect of amitriptyline appears to be ASM-independent. Detailed changes in signalling pathways need be investigated in upcoming studies.

It is remarkable that amitriptyline 5 µM alone has the same effect as the potent combination of IBMX (a phosphodiesterase inhibitor) and salbutamol on bronchodilatation and inhibition of recontraction of the airways. Perhaps, these differences depend on various signalling pathways, but also on different durations of action. Using methacholine as a bronchoconstrictor with prolonged action, we observed constant airway contraction 120 min after stimulation. However, after 120 min the bronchodilatory effect of amitriptyline was also still measurable in dilatated slices (data not shown), while the effect of IBMX and salbutamol had decreased. Further studies should therefore investigate whether amitriptyline can be used as an alternative to ß_2_-antagonists such as salmeterol with prolonged effects.

In summary, we have demonstrated in in-vitro and in-vivo experiments that amitriptyline is a drug of high potential for inhibition of acute bronchoconstriction and induction of bronchodilatation of pre-contracted bronchi. Further studies, particularly into the immunological effects of amitriptyline, are warranted since it appears to be the first therapeutic agent in asthmatic disease to have strong effects on the T_H_2-allergic phenotype and on acute bronchoconstriction. Administration of this drug through inhalation helps to reduce its typical side effects.

## Supplementary Information

**Additional file 1: Figure S1.** Amitriptyline (0.1–5 μM) is not toxic in rat PCLS. **A** LDH toxicity tests after 1h and **B** 24 h. n = 9 in all groups. **Figure S2.** Dilatation with IBMX/salbutamol and combinative therapies. **A** Minimal airway area after dilatation and stimulation with rising concentrations of methacholine. n = 4 in all groups. All graphs represent means ± SEM; *p < 0.05, ***p < 0.001.

## Data Availability

The datasets supporting the conclusions of this article are included within the article and its additional files.
