# Correction: Amitriptyline inhibits bronchoconstriction and directly promotes dilatation of the airways

**DOI:** 10.1186/s12931-025-03353-z

**Published:** 2025-09-12

**Authors:** Paulina Hempel, Virag Klein, Anna Michely, Svenja Böll, Annette D. Rieg, Jan Spillner, Till Braunschweig, Saskia von Stillfried, Norbert Wagner, Christian Martin, Klaus Tenbrock, Eva Verjans

**Affiliations:** 1 Department of Pediatrics, Medical Faculty, RWTH Aachen, University Hospital Aachen, Pauwelsstraße 30, Aachen, 52074 Germany; 2 Institute of Pharmacology and Toxicology, Medical Faculty, RWTH Aachen, University Hospital Aachen, Aachen, Germany; 3 Department of Anaesthesiology, Medical Faculty, RWTH Aachen, University Hospital Aachen, Aachen, Germany; 4 Department of Thoracic and Cardiovascular Surgery, Medical Faculty, RWTH Aachen, University Hospital Aachen, Aachen, Germany; 5 Institute of Pathology, Medical Faculty, RWTH Aachen, University Hospital Aachen, Aachen, Germany

**Correction: Respir Res 24, 262 (2023)**

**https://doi.org/10.1186/s12931-023-02580-6**

In the original publication of this article [1], the Author contributions statement was incorrectly given as “Writing and correction of the paper: PH, EV and CM“ but should have been “Writing of the paper: PH. Correction of the paper: PH, EV, and CM“. The authors apologize for this error and state that this does not change the scientific conclusions of the article. The incorrect and correct version of Author Contributions are displayed below. The original article has been updated.

Incorrect:

Author contributions

Conception and design: PH, EV, NW, CM and KT; performed research and pre-studies: PH, VK, AM, EV, AR; analysis and interpretation: PH, VK, AM, EV, KT and CM; contributed reagents/materials/tissue specimens: EV, JS, SS, TB, KT, CM; writing and correction of the paper: PH, EV and CM.

Correct:

Author contributions

Conception and design: PH, EV, NW, CM and KT; performed research and pre-studies: PH, VK, AM, EV, AR; analysis and interpretation: PH, VK, AM, EV, KT and CM; contributed reagents/materials/tissue specimens: EV, JS, SS, TB, KT, CM; writing of the paper: PH. Correction of the paper: PH, EV, and CM.

## References

[1] Hempel P, Klein V, Michely A, et al. Amitriptyline inhibits bronchoconstriction and directly promotes dilatation of the airways. Respir Res. 2023;24:262. 37907918 10.1186/s12931-023-02580-6 PMC10617234

