# Supplementary material for: Amitriptyline inhibits bronchoconstriction and directly promotes dilatation of the airways
Source: Respir Res. 2023 Oct 31;24:262. doi: 10.1186/s12931-023-02580-6 (PMC10617234; doi:10.1186/s12931-023-02580-6)
Supplement: Supplementary file 1 — Additional file 1: Figure S1. Amitriptyline (0.1–5 μM) is not toxic in rat PCLS. A LDH toxicity tests after 1h and B 24 h. n = 9 in all groups. Figure S2. Dilatation with IBMX/salbutamol and combinative therapies. A Minimal airway area after dilatation and stimulation with rising concentrations of methacholine. n = 4 in all groups. All graphs represent means ± SEM; *p < 0.05, ***p < 0.001. [file 12931_2023_2580_MOESM1_ESM.pdf]

Fig. S1

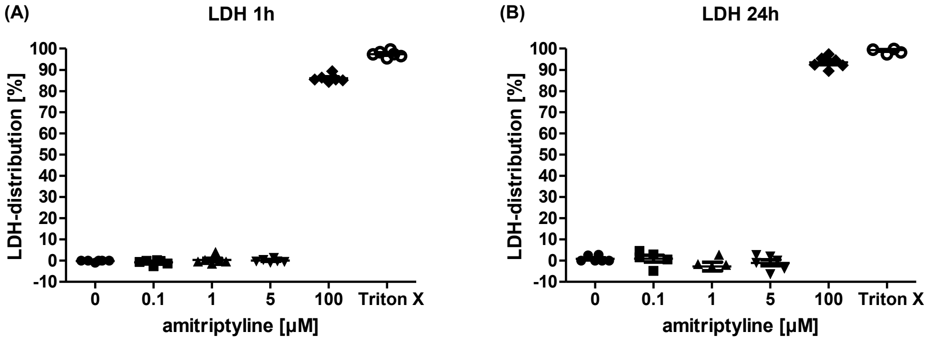

Supplemental Fig. 1: Amitriptyline (0.1-5µM) is not toxic in rat PCLS.
(A) LDH toxicity tests after 1h and **(B)** 24h. n=9 in all groups.

Fig. S2

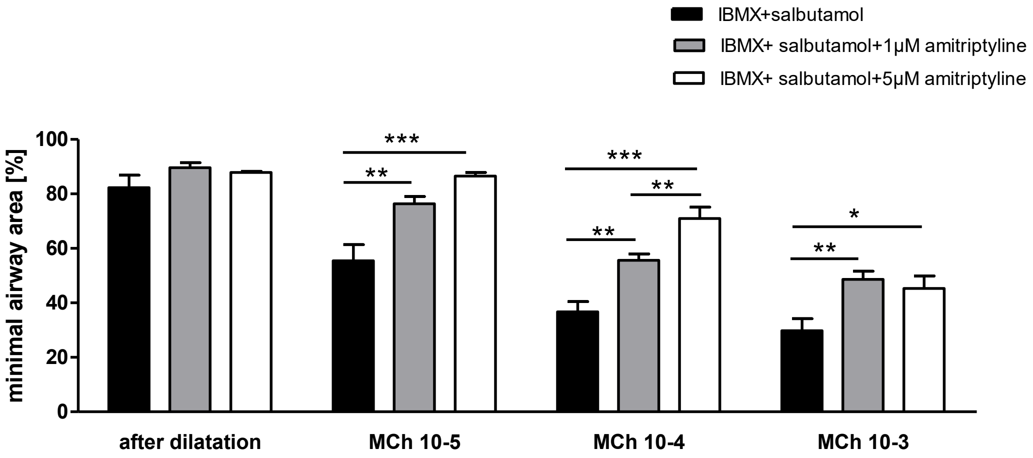

Supplemental Fig. 2: Dilatation with IBMX/salbutamol and combinative therapies. (A) Minimal airway area after dilatation and stimulation with rising concentrations of methacholine. n=4 in all groups. All graphs represent means +- SEM; *p < 0.05, ***p<0.001.
